# Multiple Strategies for Spatial Integration of 2D Layouts within Working Memory

**DOI:** 10.1371/journal.pone.0154088

**Published:** 2016-04-21

**Authors:** Tobias Meilinger, Katsumi Watanabe

**Affiliations:** 1 Research Center for Advanced Science and Technology, The University of Tokyo, Tokyo, Japan; 2 Department for Human Perception, Cognition and Action, Max Planck Institute for Biological Cybernetics, Tübingen, Germany; 3 Department of Intermedia Art and Science, Waseda University, Tokyo, Japan; Durham University, UNITED KINGDOM

## Abstract

Prior results on the spatial integration of layouts within a room differed regarding the reference frame that participants used for integration. We asked whether these differences also occur when integrating 2D screen views and, if so, what the reasons for this might be. In four experiments we showed that integrating reference frames varied as a function of task familiarity combined with processing time, cues for spatial transformation, and information about action requirements paralleling results in the 3D case. Participants saw part of an object layout in screen 1, another part in screen 2, and reacted on the integrated layout in screen 3. Layout presentations between two screens coincided or differed in orientation. Aligning misaligned screens for integration is known to increase errors/latencies. The error/latency pattern was thus indicative of the reference frame used for integration. We showed that task familiarity combined with self-paced learning, visual updating, and knowing from where to act prioritized the integration within the reference frame of the initial presentation, which was updated later, and from where participants acted respectively. Participants also heavily relied on layout intrinsic frames. The results show how humans flexibly adjust their integration strategy to a wide variety of conditions.

## Introduction

In daily life, people experience rooms, buildings or neighborhoods, but also information on displays from successive gazes and views. Although they might never see the whole environment at once, they nevertheless develop a grasp of its overall structure. To form this, observers must spatially integrate the separately experienced spatial information into a common reference frame [1–3]. The underlying processes are still widely unknown.

At least two levels of spatial integration can be distinguished: integration across gazes into a common view and integration across views. For example, when learning an object layout from a single viewpoint, multiple eye fixations may eventually be integrated into a common view. Typically, visual information largely overlaps between gazes. Such overlap is not necessarily present when integrating multiple views, for example, opposite room views. Furthermore, integration across gazes happens on a short time scale and is usually examined within 2D screens [4–6]. Integration across views may happen in 2D (e.g., on a screen) and in 3D, both on a short and long time scale (e.g., when moving within a room or when revisiting a room). The present study examined view integration in 2D within working memory taking into consideration the results on spatial integration obtained from 3D short- and long-term learning [1,3,7–10].

Unless given incentives and sufficient time to integrate beforehand, spatial integration is mentally costly: performance is better when acting on separately learned spatial layouts compared to acting on the combination of the two layouts [11–15]. One part of integration costs involves transforming misaligned spatial information into a common reference frame [3].

One key issue in the integration of spatial information is the question of which reference frame (i.e., coordinate system) is used for integration. For integration across gazes, retinal and non-retinal (i.e., head-, torso-, or environment-based) reference frames may be used. Despite the subjective constancy of our surrounding world, it has been shown that the whole visual field is not automatically integrated across gazes [16]. However, adaptations to line orientation, form, or faces persist across gazes [17], thus indicating the automatic updating of certain attended features across gazes. Visual landmarks are used as an environmental reference to locate objects across gazes [5,6]. If sufficient time is given, participants can integrate object locations across gazes within a single view and memorize it [2].

When integrating across views, multiple solutions for integrating reference frames are possible. For example, when learning two misaligned layouts (layout 1 and layout 2) integration could happen in the reference frame of layout 1 or layout 2, or in the reference frame from which the information is used afterwards. Additionally, an independent reference frame might be used; for example, one along a very salient orientation, such as the main axis of the surrounding room. Prior research showed indications of all four cases [1,3,7–10]. Why would participants use such a wide variety of reference frames for integration? Prior examinations differed considerably in their methodology using different stimuli, learning time, or providing the possibility to update information between presentations. The present study aim is to construct a single setup within which these factors could be isolated from each other, varied, and tested for their responsibility of triggering a certain reference frame for integration. In the following, we will review prior research, isolate potentially crucial factors, describe a setup within which these factors can be tested, report on experiments that examined these variations, and discuss their implications.

Evidence for integrating within the reference frame of earlier experienced information comes from studies in which participants learned layout 1 separately and, then, layout 1 and 2 together [1,7–10]. Subsequent memory tests involving both layouts required integrating information from both layouts. For example, Kelly and McNamara [8] had participants learn a layout of objects on the floor. Afterwards, a second layout was added and both were learned together from the same or from a different viewpoint. Subsequent imagined perspective-taking tasks indicated that participants used the reference frame of the first layout (i.e., its learning perspective) to also encode information from the second layout. Similar results were obtained within a virtual environment [7]. These studies also tested conditions in which the second viewpoint was more salient than the first viewpoint (e.g., because of its initial structure or it was aligned with the walls of the surrounding room). Here, spatial information might be reorganized toward the reference frame of the second viewpoint [7,8]. Participants also used the orientation of the overall layout for reference (i.e., the form of layout 1 and 2 seen together) rather than the intrinsic orientation of the individual layouts when learning from one viewpoint [1].

Results suggest that participants used a reference frame established on prior information for encoding subsequently added information unless another reference frame was very salient in which case it was used instead (e.g., the main intrinsic orientation of the overall layout or the surrounding room). One characteristic of these tasks is familiarity. Participants learned object layouts in the order of minutes, which were usually terminated only after reaching a criterion level in a pointing task. Therefore, participants extensively familiarized themselves with the layout and sometimes also the pointing task. This familiarity likely yielded a well-established memory trace and only afterwards did they experience additional information with the prior layout always visible. As a first goal, we examined whether familiarity with a task together with familiarity with the layout as established by the self-determined learning time would result in prioritizing earlier reference frames.

In the aforementioned experiments, participants used earlier established reference frames or later salient ones. Salient orientations may be ones aligned with a grid layout easy to verbalize, for example, by rows and columns, [18], or aligned with the surrounding walls [19]. The opposite case of excluding any salient orientations that could provide a reference frame for integration is rather difficult, if not impossible. Therefore, in prior studies, salient orientations were either earlier presented information or later presented information. To account for this shortcoming, secondly, we aimed to balance salient layout orientations with the use of earlier and later reference frames aligning the layout equally often with each reference frame.

Another characteristic of prior studies is that both layouts could be learned together during the second presentation when both were visible. However, often, relevant spatial information cannot be perceived at the same time; for example, when integrating the front and back view of a room, a house, or views of multiple rooms. These views are experienced separately and must be brought together without profiting from having them present within a single view. Our third goal, therefore, was to examine whether prior results also generalize to spatial integration of separate experiences.

Prior experiments indicated the use of earlier and salient later reference frames. However, one study also suggested the integration within the reference frame in which participants acted [3]. In this study, participants were required to plan and walk the shortest path across floor tiles that lit up briefly during two presentations. Between presentations, participants changed their viewpoint by walking around the tiles. Participants were thus able to update the reference frames of prior experiences to their later viewpoint (i.e., they could memorize the tiles relative to their body and actualize these locations while walking around the tiles). Participants integrated the two views upon the lit-up tiles in the reference frame in which they conducted the task (i.e., in which they started walking across the tiles). This acting reference frame was either the frame of the second presentation or it was the viewpoint of the first presentation when they walked back to it before acting. It remains unknown why participants used the acting reference frame for integration. Did they do so because they always updated all spatial information and did so until they acted?, or because they knew beforehand within which reference frame they acted afterwards and, thus, transformed the spatial information into this reference frame, maybe even during the presentation of the tiles. As the fourth goal both possibilities were examined within the present study. We also disentangled the reference frames of acting from the first and second presentation.

In order to examine these questions, we conducted four experiments in which participants were required to integrate separate layout parts. Experiment 1 acted as a baseline. Here, we wanted to see which reference frames participants spontaneously used. Experiment 2–4 examined whether this baseline pattern could be influenced by familiarity, updating, and knowing within which orientation to act afterwards. In Experiment 2, participants familiar with the task could self-determine how long they watched each stimulus. We expected them to prioritize the reference frame of earlier presented information. In Experiment 3, the layout visibly moved and rotated from its position during the first presentation to its position and orientation during the second presentation. The reference frame orientation of the first presentation could be updated to the orientation of the second presentation. Here, we expected prioritizing the reference frame of the later presentation. In Experiment 4, participants knew beforehand in which reference frame orientation they had to act. Here, we expected reliance on the acting reference frame.

## Experiment 1: Baseline

Experiment 1 was conducted to obtain a baseline for spatial integration strategies in our spatial integration task.

### Methods

#### Participants

A total of 16 women and 21 men participated in the experiment. They were on average 21.6 years old (*SD* = 3.1). Three additional participants (two women) were not significantly better than the chance rate (see below) and were not included. Participants were recruited through a university participant panel. They gave written informed consent before conducting the experiment and were paid for their participation. The experimental procedure was approved by the institutional review board of The University of Tokyo.

#### Materials and tasks

**Fig 1:**
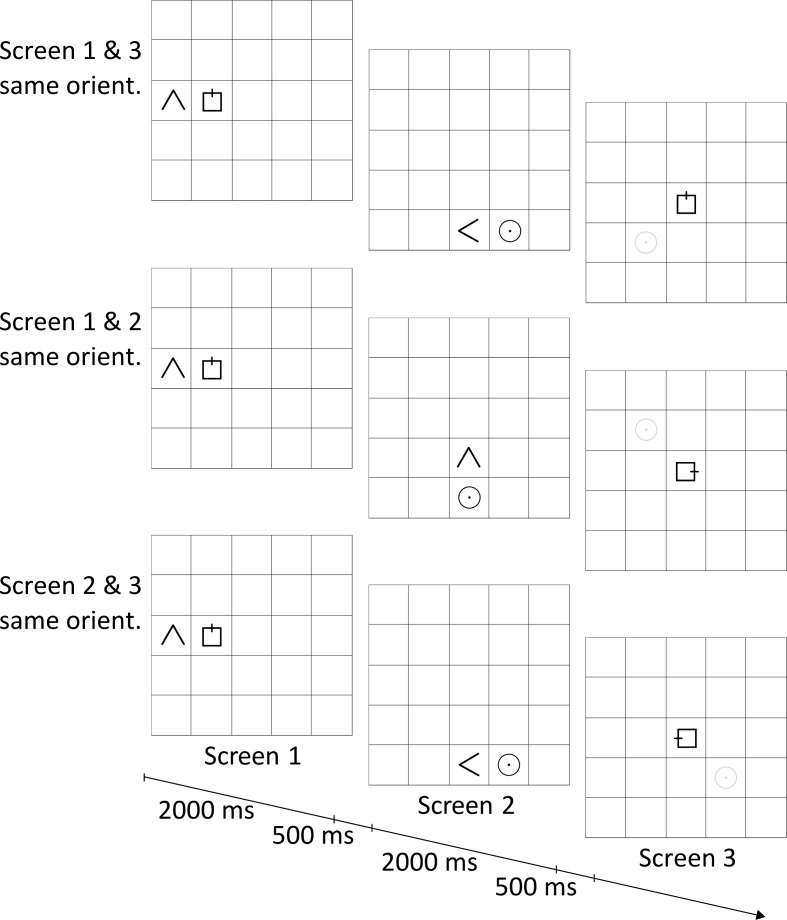
Illustration of three example trials used in the experiment. In screens 1 and 2, participants saw two parts of a layout. They integrated these parts to indicate the location of the circle relative to the rectangle presented in the center of screen 3. The correct location is displayed by the grey circle, which was not visible in the experiment. The presented layouts had the same orientation in all, none, or two out of the three screens. For example, in the top line the layout is oriented upwards in screen 1 and 2, but to the left in screen 2. The timeline at the bottom indicates presentation times for each screen and for a blank white screen in between. In screen 3, we measured the time until participants clicked the grid cell in which they located the circle.

As illustrated in Fig 1, the participants' task was to determine the form of a layout of three objects that were presented in two parts on a 5 × 5 cell grid. The layout consisted of a “∧” sign, here called triangle, a rectangle with a small bar perpendicular to one of its sides, and a circle with a dot at the center. The three objects formed an L-shape with the rectangle placed left or right of the triangle and the circle above or below the triangle. A trial consisted of three screens within which the symbols were displayed. In screen 1, the rectangle and triangle were visible; in screen 2 the triangle and circle; and, in screen 3, only the rectangle. Participants used the triangle in screens 1 and 2 to infer the location of the circle relative to the rectangle. For this, they had to take the orientation of the triangle into account as the overall layout might have rotated between screens. Between screens 2 and 3, it might have rotated again. In screen 3, a mouse pointer appeared in the middle of the rectangle and participants were asked to click on the grid cell containing the circle using the mouse. We measured latency and correct responses.

We used three objects as the minimum number of objects, which allows us to examine the integration of two separate presentations. With the grid, we could determine exact responses (i.e., indicating the correct or a wrong grid cell) and also assign eye fixations concurrently as measured throughout each trial. A layout with objects not adjacent could have posed extra difficulty, which we avoided. Three objects in a row are adjacent to each other as well. However, to not add variation to the form of the layout, we only used the L-shape. Due to the L-shape, the circle was always either in the cell left-above, left-under, right-above, or right-under the rectangle. Therefore, the chance level was defined as 25%. This is a conservative estimate as participants could click anywhere on the screen, which would have resulted in a much lower chance level. However, the chance rate was only used to identify participants with major problems in the task and, for this, 25% seemed more realistic.

#### Intrinsic orientation

In screen 1, the rectangle and triangle were always displayed next to each other at one side of the grid (i.e., left as in Fig 1, or right, top, or bottom). Throughout the experimental trials, their relative orientation was always as displayed in Fig 1. Although they always pointed in the same direction, the common direction varied relative to the screen. Thus, in screen 1 they jointly pointed to the top, as in screen 1 of Fig 1, or to the right, left, or bottom. The triangle was equally often left and right of the rectangle, and the circle was equally often above and below the triangle. We did not change the relative orientation of the rectangle and triangle to each other. As indicated in the test experiments, that would have been too demanding for many participants. As a consequence, the overall layout showed a clear intrinsic orientation throughout all of the experiment’s trials.

Please note that each trial consisted of learning a layout. In many prior experiments, participants learned only a single overall layout and their acquired knowledge was tested multiple times. The present approach has the advantage in that salient intrinsic orientations can be balanced with earlier, later, and acting reference frames.

#### Orientation match conditions

As displayed in Fig 1, layout presentations between two screens coincided or differed in orientation. We examined all five possibilities of matching and non-matching orientations between the three screens: all three screen orientations matched (condition “all same”), all screen orientations differed (condition “all different”), or the orientations of two screens matched each other, but not with the third screen: screens 1 and 3 had the same orientation, but differed from the orientation of screen 2 (condition “1 & 3 same” see Fig 1, first line); screens 1 and 2 had the same orientation, which differed from the orientation of screen 3 (condition “1 & 2 same” see Fig 1, second line); or screens 2 and 3 had the same orientation, which differed from the orientation of the layout in screen 1 (condition “2 & 3 same” see Fig 1, third line). Participants had to integrate the differently oriented layout parts within a single reference frame in order to fulfill the task. To do so, differently oriented presentations had to be aligned with each other. This alignment could happen in different reference frames: the reference frame within which earlier information was presented (i.e., the reference frame of screen 1 or RF1), the reference frame where later information was presented (i.e., the reference frame of screen 2 or RF2), or the reference frame within which participants acted (i.e., the reference frame of screen 3 or RF3). Irrespective of the reference frame in which the two layouts were integrated, the integrated layout had to be transformed to the reference frame of screen 3 from which the answer was given. This potentially required transformations to align the layout parts for integration and for giving answers. Aligning misaligned spatial information is known to increase errors/latencies [2,3] and the required translations were identical in all orientation matched conditions. The error/latency pattern between the five conditions was indicative of the required alignment costs and of the reference frame used for integration.

**Fig 2:**
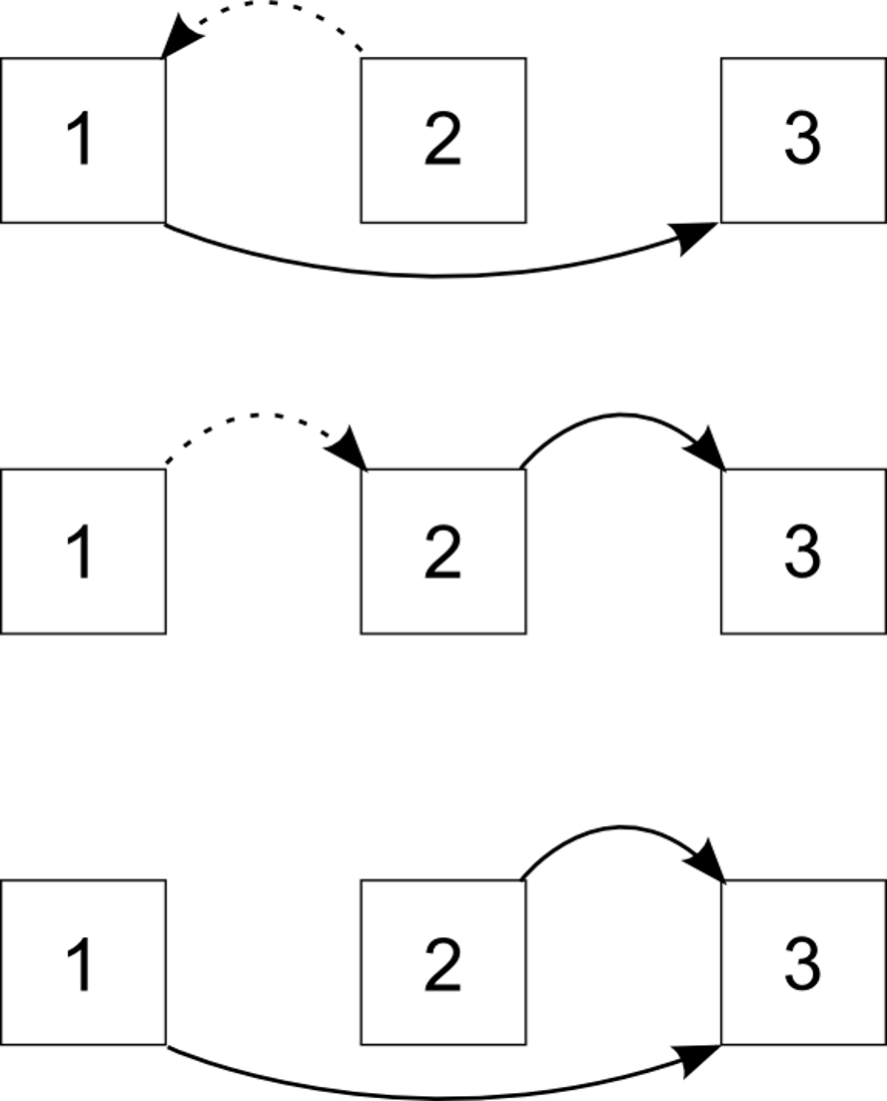
Illustration of required reference frame transformations. Spatial information presented in screen 1 and 2 is integrated within the reference frame of screen 1 (top), screen 2 (middle), or screen 3 (bottom). Regardless of which reference frame participants integrated, they always reacted on the integrated layout in screen 3. Arrows indicate required transformations. Transformation costs for dotted arrows might be negligible due to updating or sufficient time.

The following predictions are illustrated in Fig 2. When integrating spatial information in the acting reference frame (i.e., RF3, Fig 2 bottom line), information from RF1 and RF2 must each be rotated into RF3. If RF3 is identical to RF1 and RF2 in the “all same” condition, no rotation costs occur and participants should perform best. One rotation is required if RF3 is identical to either RF1 or RF2 as in the “1 & 3 same” and “2 & 3 same” conditions. Participants should perform the second best. Two rotations are required if RF3 is different from both RF1 and RF2 as in the “1 & 2 same” condition and in the “all different” condition; participants should perform worst. These transformations can occur only within screen 3, as participants know RF3 orientation only after screen 3 onset. Therefore, the pattern is predicted for both errors and latency: all same < 1 & 3, 2 & 3 < 1 & 2, or all different

When integrating within RF1 in which earlier information was presented, information from RF2 must be transformed into RF1 and, from there, into RF3 (Fig 2 top row). Conditions in which RF1 and RF2 as well as RF1 and RF3 are identical should profit, yielding the following error/latency pattern: all same < 1 & 2, 1 & 3 < 2 & 3, or all different. To integrate in the later RF2, information from RF1 is transformed into RF2 and, from there, into RF3 (Fig 2 middle row). Conditions in which RF1 and RF2 and RF2 and RF3 are identical should profit, yielding the following error/latency pattern: all same < 1 & 2, 2 & 3 < 1 & 3, or all different.

#### Rotation center conditions

**Fig 3:**
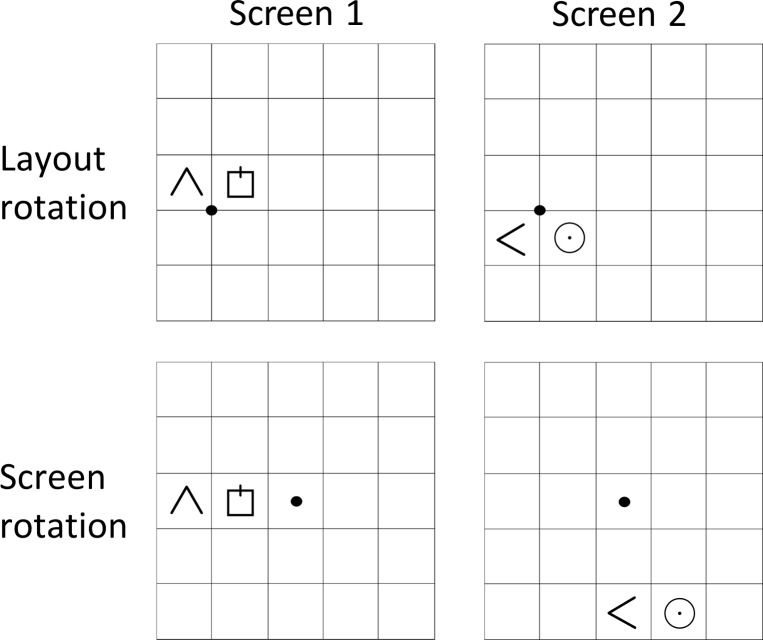
Illustration of layout and screen rotations. Between screens 1 and 2, the presented layout rotated either around the grid cross between the three layout objects or around the center of the screen. Rotation center points are indicated by the black dots, which were not visible during the experiment.

From screen 1 to screen 2, the layout either rotated 90° clockwise, 90° counterclockwise (as in Fig 1), or it did not rotate at all. As displayed in Fig 3, the layout rotated either around the screen center (i.e., the middle cell of the grid; “screen rotation”) or rotated around the center of the layout (i.e., the grid point between the rectangle, triangle, and circle; “layout rotation”). The motivation for this variation was to ascertain whether participants used screen-relative coordinates or layout-relative ones (i.e., where the origin of their reference frame was located). In case participants used screen-coordinates, rotation around the screen center should be easier. If they relied on layout-based reference frames, rotation around the layout center might have been easier. In case of no rotation, the layout stayed either at the same spot, which worked as a control condition for “layout rotation,” or the layout moved to the location where it would be after rotating around the screen center, only without rotating. From screens 2 to 3, the layout always moved from its location at one side of the screen to the center. In neither case was an object presented at a location where another object was presented on the screen before.

#### Condition balancing

The main variation of the experiments was the orientation match. A pairwise balance was used with rotation center, layout form (i.e., which of the four layouts was used), layout orientation at the start (i.e., whether the rectangle and triangle pointed upwards, downwards, or to the left or right), and rotation direction (clockwise vs. counterclockwise between screens 1 and 2). From all possible trials, a random subset of 60 trials were chosen, which fulfilled these balancing constraints. We used these 60 trials in the experiments.

#### Timing

A trial started with a fixation cross presented for 1500 ms. As also displayed in Fig 1, stimuli in screens 1 and 2 were presented for 2000 ms each. This duration ensured that participants had sufficient time to encode the stimuli into working memory as we were not interested in encoding processes [20–22]. Between stimuli presentations, participants saw a blank screen for 500 ms. If participants did not react within 10 seconds at screen 3, this was considered a miss and the next trial was started. The next trial always followed immediately after the previous one.

#### Setup and procedure

Participants sat in front of a CRT monitor. The experiment was presented on a rectangular 29 × 29 cm area in the center of the monitor screen with a resolution of 1024 × 1024 pixels. Participants put their heads on a chinrest so their eye height was in the middle of the screen 58 cm away. The experiment ran on a MacBook Pro with Matlab using the Psychophysics and Eyelink toolbox extensions [23]. The code is available upon request.

Participants received written and verbal instructions. They trained for the task for as long as they wanted on a different set of trials. During training, the experimenter ensured that participants understood the task. Then, the eye tracker was calibrated and the experiment started. Trials followed each other and were presented in a random order that was determined individually for each participant. Participants were instructed to react as quickly and accurately as possible. After the experiment, participants completed a questionnaire asking their age, subjective sense of direction, and whether they applied certain subjective strategies (e.g., verbalizing the layout or mentally rotating it). The overall procedure lasted about 30 min.

#### Eye tracking

We recorded eye fixations within single grid cells along a trial using an individually calibrated Eyelink 1000 running at 500 Hz. The automatic fixation extraction provided by the software offered from SR research was used. We employed an extraction setting optimized for cognitive experiments, which pools micro-saccades into longer fixations. If a fixated cell was occupied by a layout object—currently visible or not—the fixation was assigned to this object. For example, looking at the correct location of the non-visible circle in screen 3 was considered a circle fixation. Within each screen, we analyzed fixation sequences across objects, ignoring fixations at non-object locations and multiple subsequent fixations at the same location. In the following, we describe fixation patterns from Experiment 1, which were largely representative also for the other experiments.

In screen 1, the rectangle and triangle were displayed. Most participants either fixated on only the rectangle (27% of the cases), looked then at the triangle (24%), or continued going back (31%) and sometimes forth again (7%). Rarely, participants looked only at the triangle (4%) or at the triangle and then the rectangle afterwards (2%).

In screen 2, the triangle and the target circle were visible. Sequences included looking only at the triangle (16%), circle (14%), or continuously looking between the two (27%). In 43% of the cases, participants initially (8%) or eventually looked at the correct location of the rectangle, which was not displayed. This suggests that, at least in some cases, participants had determined the whole layout already in screen 2. However, rectangle fixations were not associated with higher accuracy or faster reactions afterwards, *F*s < 1.

In screen 3, the rectangle was always displayed in the center of the screen, but its orientation differed, which was crucial for the task. In most cases, participants looked at the rectangle first, and then continued looking at the correct location(s) of the triangle and/or the circle (40%) or looked at other screen locations (37%) where they might have assumed the layout. Looking at correct (non-visible) layout locations was associated with fewer errors, *F*(1, 1574) = 57.6, *p* < .001, and quicker reaction times, *F*(1, 1293) = 36.7, *p* < .001, looking at other locations corresponded with higher error rates, *F*(1, 1571) = 136, *p* < .001, and latencies, *F*(1, 1290) = 102, *p* < .001. Participants seemed to have looked at non-visible layout locations. Those with the wrong conception of the layout might have been more unsure about the target location and thus took longer to react. Sometimes, participants first gazed at the (correct) locations of the triangle (13%) or the circle (10%). However, as we recorded only the first new fixation location after the onset of screen 3, participants might have looked at the upcoming location of the rectangle even before it was displayed and continued from there to their “first” fixation on the triangle or circle. Notably, only in 0.1% of the cases did participants look only at the rectangle and nowhere else on the screen. The tasks were not solved independent of eye-fixations at task-relevant locations. The overall pattern was also similar in the other experiments. We did not find stable differences in fixation patterns, fixation frequency, or duration across experiments as a function of experimental variations.

#### Data analysis and design

Not reacting within 10 s (average 0.08–0.36% per experiment) and clicking on the wrong grid cell were considered as error responses. If a participant’s hit rate was not significantly higher than the chance rate of 25% (defined as randomly guessing one of the four grid cells cornering the rectangle in screen 3), their data were not analyzed. We used latency data from correct trials and deleted values deviating more than 3 SD from the overall mean (1–2% per experiment).

Errors and latencies were submitted to a linear mixed model analysis with the within-participants factors for orientation match (5 levels) and rotation center (screen vs. layout). Each of the 10 conditions was repeated 6 times. We used planned pairwise comparisons between the orientation match conditions to examine the predicted patterns. Observing the multiple predicted differences of a pattern in random data is highly unlikely. As predictions were correlated (e.g., all patterns predicted best performance for the “all same” and worst performance for “all different” condition), pairwise comparisons between conditions also more clearly differentiated between the individual patterns than overall similarity with a predicted pattern. For example, condition “1 & 3 same” is predicted to show better performance than “2 & 3 same” when integrating in early reference frame, worse performance when integrating in later reference frame, and no difference when integrating in the reference frame of acting.

In order to estimate the use of the layout intrinsic reference frames, we compared layout orientations within screen 3 with each other using a within-participant linear mixed model analysis (4 orientations). A layout orientation with a rectangle (and triangle) pointing upwards as in screen 1 of Fig 1 was arbitrarily defined as 0°. The layout in screen 3 in the first line of Fig 1 was along the intrinsic layout orientation, while the orientations in screen 3 lines 2 and 3 were not. Full-factorial crossing of test orientation with orientation match and rotation center would have resulted in unequal cell numbers. Crossing was also not possible in Experiment 4, but we wanted to conduct the same analysis in each experiment. Therefore, we did not use full factorial crossing of these three factors. When conducting the analysis in Experiments 1–3, the resulting patterns were highly similar.

Compared to an ANOVA, linear mixed model analysis is less restrictive regarding distribution assumptions [24]. Commonly accepted effect sizes for linear mixed models are not yet available. Thus, we report partial eta squares (η_p_^2^) derived from data aggregated per participant and the respective condition. Unless otherwise explicitly mentioned, all significant results at *p* < .05 are reported.

All relevant data are within the supporting information S1 Dataset.

### Results

#### Early, late, and acting reference frames

**Fig 4:**
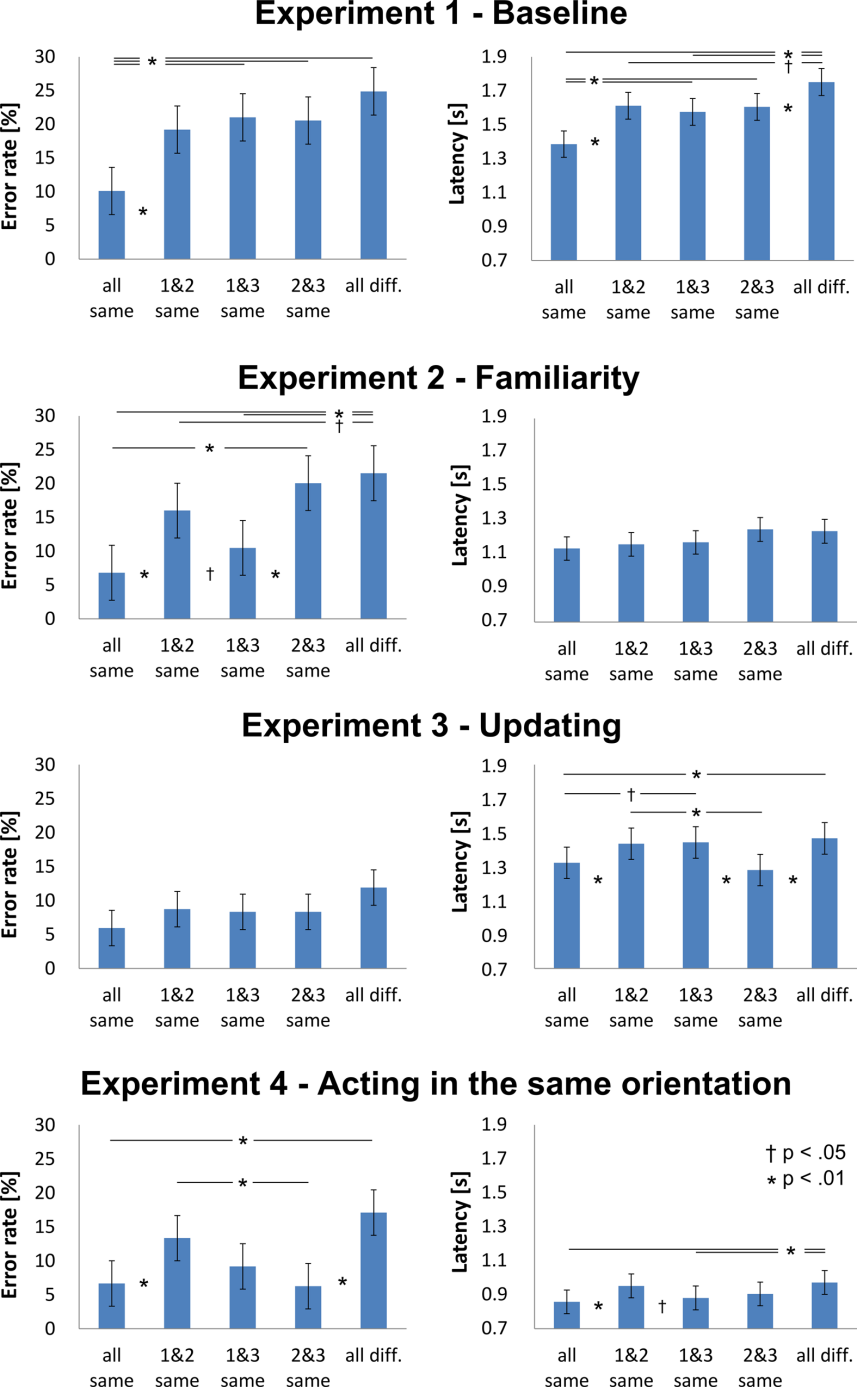
Reference frames used for integration as indicated by orientation match conditions. Mean error rate (left), latency (right), and standard errors as estimated from the marginal means are displayed. Asterisks and daggers indicate significant differences in pairwise comparison.

As shown in Fig 4 (top row), the mean error rates, *F*(4, 2154) = 11.1, *p* < .001, η_p_^2^ = .12, and mean latencies, *F*(4, 1696.3) = 13.0, *p* < .001, *η*_*p*_^*2*^ = .15, differed depending on which screen orientations matched. For latency, this was qualified by an interaction with the rotation center, *F*(4, 1694) = 4.04, *p* = .003, η_p_^2^ = .10; this did not change any main effect of the orientation match. Planned pairwise comparisons showed that performance in the “all same” condition was quicker, *F*s > 17.7, *p*s < .001, η_p_^2^ > .15, and more accurate compared to in the other conditions, *F*s > 11.7, *p*s < .002, η_p_^2^ > .13. When the case layout orientations differed in all screens, participants responded slower than they did when at least two orientations coincided, *F*s > 6.73, *p*s < .010, η_p_^2^ > .06. The results indicated that the participants were sensitive to the amount of rotation between screens. However, no clear strategy as predicted was observed. A visual inspection of the individual performance patterns suggests that roughly equal proportions of participants showed patterns resembling integration within earlier, later, as well as acting reference frames. While this is not a statistically reliable assignment it suggests that there was some variability in how individual participants solved the task.

#### Layout intrinsic reference frames

When looking at layout intrinsic alignment effects, there was a strong effect of layout orientation during testing on latency, *F*(3, 1700) = 42.4, *p* < .001, η_p_^2^ = .39. When the rectangle was presented upright (cf., the orientation in screen 3, top line of Fig 1) participants reacted quicker than they did when the rectangle pointed to the left or right, *F*s > 14.0, *p*s < .001, η_p_^2^ > .18, which was quicker than when the rectangle pointed downwards, *F*s > 39.4, *p*s < .001, η_p_^2^ > .35. The more the layout orientation deviated from being upright, the slower the participants reacted. This indicated that the participants strongly relied on layout intrinsic reference frames.

### Discussion

The participants in Experiment 1 relied on layout intrinsic reference frames. This is in line with prior integration experiments [7,8] and spatial learning in general (see McNamara et al., 2008 for an overview) in which salient intrinsic reference frames were widely used if present. This replicates work from learning object layouts within a room to learning layouts presented on a screen for a much shorter time. Importantly, the present results show that intrinsic reference frames play a role not only in experiments in which a single layout was learned or where layout 1 was learned first, and then layouts 1 and 2 were learned together. Present results show that intrinsic reference frames also matter when integrating separate experiences never seen together within a single view.

Participants were sensitive to the amount of layout rotations conducted throughout a trial. However, there was no clear prioritizing of reference frames within which to integrate information: earlier, later, or acting reference frames. Individual participants seemed to rely more on specific strategies. If participants exclusively relied on intrinsic reference frames, no effect of layout rotations would have been observed.

The results of Experiment 1 provided a baseline for further comparisons. The setup itself did not prioritize integration in early, late, or acting reference frames as such. In the subsequent experiments (Experiments 2 to 4), we changed the circumstances in a way to introduce such prioritizing based on the considerations established in the introduction.

## Experiment 2: Familiarity

Preferences for early reference frames were all found in experiments in which participants learned object layouts within minutes rather than seconds [7–10]. Their acquired knowledge was often tested for accuracy before they proceeded to the test phase. Consequently, participants were familiar with the task and layout. We introduced these circumstances to the present setup to see whether participants would now preferably integrate in the earlier reference frame as well.

### Methods

We used participants who had familiarized themselves sufficiently with the task at hand. This was done by using only participants from Experiment 1. Ten women and thirteen men had a short break after Experiment 1 and then proceeded to Experiment 2. They were 21.6 years old on average (*SD* = 3.3). Due to this approach, participants were familiar with the task’s properties. Furthermore, to familiarize themselves with the layout, participants were granted as much learning time in screens 1 and 2 as they wanted. Participants pressed the space bar on a keyboard to proceed to the next screen. They were instructed that they could take as much time as they wanted before pressing the space bar. However, just as before, they should react as quickly and accurately as possible within screen 3. Participants used both hands: their dominant hand to operate the mouse and the other hand to press the space bar. Everything else, including the trials used, was identical to Experiment 1.

We predicted that participants would integrate within the early reference frame of screen 1. Due to the self-paced learning time, participants could largely compensate for the transformation costs for aligning the layout between screens 1 and 2. Thus, the dotted arrows in Fig 2 do not matter, while only the rotation costs from RF1/RF2 to RF3 are relevant (solid arrows). The predictions for integrating in RF1 or RF2 simplify the predicted patterns. When integrating within RF1 only the transformation from RF1 to RF3 are relevant. This transformation must occur during presentation of screen 3 and is time pressured. The cost should show up in errors and/or latency. In the “all same” and “1 & 3 same” conditions, RF1 and RF3 are identical and no such costs apply. In these conditions, participants should perform faster and more accurately than in all other conditions where such a rotation is required resulting in the following pattern: all same, 1 & 3 < 1 & 2, 2 & 3, or all different. The situation is similar when integrating in RF2. Ignoring transformation costs from RF1 to RF2, only rotation from RF2 to RF3 is required. Then, participants should perform better in the “all same” and “2 & 3 same” conditions compared to the other conditions: all same, 2 & 3, < 1 & 2, 1 & 3, or all different.

### Results

#### Early, late, and acting reference frames

As shown in Fig 4 (second row, left side), the mean error rates differed as a function of which screen orientations matched, *F*(4, 1328) = 10.9, *p* < .001, η_p_^2^ = .16. As predicted by the integration in the earlier reference frames, planned pairwise comparisons showed that performance in the “all same” and “1 & 3 same” conditions was more accurate than it was in all other conditions, *F*s > 4.22, *p*s < .040, η_p_^2^ > .05.

For the mean latencies, we found an interaction between orientation match and rotation center, *F*(4, 1098) = 8.17, p < .001, *η*_*p*_^*2*^ = .26. In conditions with rotation between screens, there was no preference for layout vs. screen rotations, *F*(1, 634.8) = 1.99, *p* = .159, η_p_^2^ = .01. However, when there was no rotation (i.e., screens 1 and 2 had the same orientation), participants reacted quicker when the layout moved between screens (control for screen rotation) compared to when it was presented at the same location, *F*(1, 441.6) = 15.3, *p* < .001, η_p_^2^ = .58. This suggested that participants might not have imagined an overlay as this should have been easier with no translation in between.

#### Layout intrinsic reference frames

When looking at layout intrinsic alignment effects, there was a strong effect of layout orientation during testing on latency, *F*(3, 1104) = 71.9, *p* < .001, η_p_^2^ = .47, and a somewhat weaker effect on accuracy, *F*(3,1334) = 3.97, *p* = .008, η_p_^2^ = .17. When the rectangle was presented upright (cf., the orientation in screen 3, first line of Fig 1), participants reacted quicker compared to when the rectangle pointed to the left or right, *F*s > 37.5, *p*s < .001, η_p_^2^ > .29. Left/right orientation itself was quicker compared to when the rectangle pointed downwards, *F*s > 71.3, *p*s < .001, η_p_^2^ > .37, as well as more accurate, *F*s > 4.18, *p*s < .042, η_p_^2^ > .14. Thus, the participants again strongly relied on layout intrinsic reference frames.

#### Presentation times

**Fig 5:**
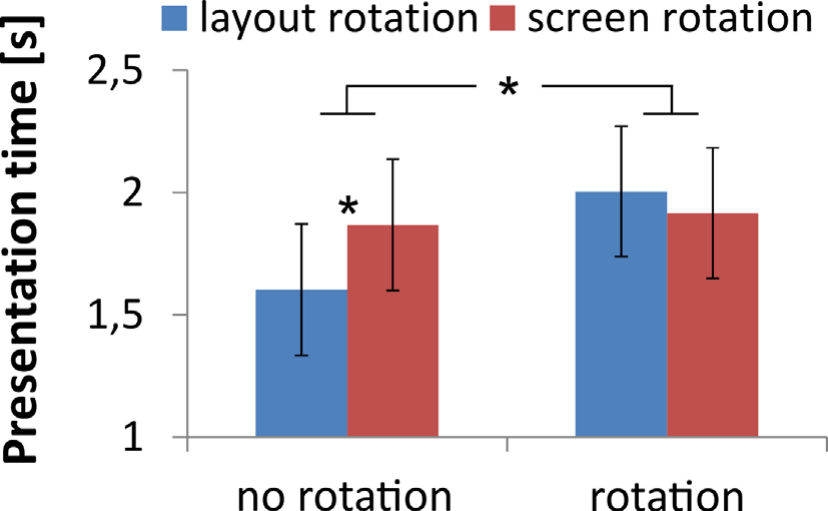
Self-paced presentation times within screen 2. “Rotation” indicates that the layout rotated between screen 1 and 2 (i.e., the conditions “1 & 3 same,” “2 & 3 same,” and “all diff”). “No rotation” indicates that the layout had the same orientation (i.e., the conditions “1 & 2 same” and “all same”). In the latter case, the layout stayed at the same screen location for (no) layout rotations or moved to another screen side for (no) screen rotations.

In Experiment 2, the participants self-selected their presentation times in screens 1 and 2. With 1.94 s (*SD* = 1.40 s) for screen 1 and 1.85 s (*SD* = 1.56 s) for screen 2, presentation times were similar to the 2 s of Experiment 1, *F* < 1. Screen 1 presentation times did not differ with orientation match or center of rotation, *F*s < 2.1, *p*s > .079. However, presentation times differed for screen 2 between orientation match conditions, *F*(4, 1309) = 5.62, *p* < .001, η_p_^2^ = .14, which interacted with the rotation center, *F*(4, 1309) = 4.36, *p* = .002, η_p_^2^ = .16. As shown in Fig 5, participants looked for a shorter period at screen 2 when there was no rotation between screens 1 and 2 (conditions “1 & 2 same” and “all same” pooled) compared to when there was a rotation (remaining conditions pooled), *F*(1, 1316) = 17.0, *p* < .001, η_p_^2^ = .17. Without rotation, participants looked for a longer period when the layout moved between the screens compared to when it remained at the same spot, *F*(1, 516) = 11.6, *p* = .001, η_p_^2^ = .31. However, looking times did not differ depending on whether the layout rotated around itself or the screen, *F*(1, 778) = 1.43, *p* = .231, η_p_^2^ = .07.

Presentation times in screen 1 differed as a function of layout orientation during screen 1, *F*(3, 1305) = 5.87, *p* = .001, η_p_^2^ = .19. Participants looked for shorter times when the layout was presented upright compared to when it was presented in other orientations, *F*s > 5.38, *p*s < .022, η_p_^2^ > .18. A similar, but less pronounced effect was found in screen 2, *F*(3, 1316) = 2.71, *p* = .044, η_p_^2^ = .08. Participants looked longer at an upside down layout than at an upright or left-pointing layout, *F*s > 4.43, *p*s < .037, η_p_^2^ > .18.

### Discussion

Participants integrated the layout in the reference frame in which they experienced the earlier presented layout part. Information was then transformed from the screen 1 reference frame to screen 3 from which it was tested. Conditions in which screens 1 and 3 had the same orientations meant that this was easier and yielded a higher observed accuracy. There was no advantage when screens 1 and 2 had the same orientation. This suggests that costs for transforming information from screen 2 into the reference frame of screen 1 were negligible. Sufficient task experience and time to do so compensated for this. Integration in the other reference frames would have resulted in different resulting patterns.

Our results parallel those from studies in which later spatial information did not replace earlier ones, but rather was added [7–10]. Prioritizing earlier reference frames is thus not limited to reference frame transfer from one layout to another, but also applies to integrating separate experiences, generalizing from 3D environments to 2D screen presentations. Task familiarity and sufficient time for integration in combination seem to largely foster earlier reference frames, which was not ubiquitously observed in Experiment 1. Contrary to the aforementioned studies, much shorter learning times, also presumably triggering working memory rather than long-term memory, can yield usage of earlier reference frames.

In the experiment task familiarity was combined with self-paced learning. Therefore, we cannot conclude whether one of these factors alone was crucial for the observed pattern not found in Experiment 1. Although learning times were similar to Experiment 1, participants’ higher task familiarity might have allowed them to encode the layout more efficiently within the same time compared to participants from Experiment 1. Self-paced learning might have mattered even if the observed learning times did not differ between experiments.

In addition to the early reference frames, participants also widely used layout intrinsic reference frames just as in Experiment 1. The closer the test orientation was to being upright, as defined by the rectangle and triangle pointing upwards, the quicker participants reacted and looked for a shorter period at screen 1. This is in line with prior studies in which salient intrinsic reference frames overrode earlier ones, if present [7,8].

The pattern of earlier reference frames was only found in accuracy, but not in latency. As participants were trained in the task, participants could have adopted a rhythm within which to act. Such a constant rhythm would eliminate latency differences between conditions; differences would only show up in errors. Similar presentation times between Experiment 1 and 2 as well as a lower standard deviation of latencies in Experiment 2 point in this direction.

Self-paced learning and prior training together guided participants toward using earlier reference frames for spatial integration. Such a strategy was not prominent in Experiment 1. Experiment 3 examined whether the updating of reference frames influenced reference frame selection in a different way.

## Experiment 3: Updating

Evidence for integrating in acting reference frames was obtained in experiments in which participants knew from where to act afterwards and in which they were able to update reference frames between presentations [3]. In order to examine how updating alone influences reference frames used for integration, we added visual updating cues to the basic setup of Experiment 1.

### Methods

After the presentation of screen 1, the screen turned white except for the triangle. Within the following 500 ms, the triangle smoothly moved across the screen to its position in screen 2. If screens 1 and 2 differed in orientation, the triangle also rotated during that time. Immediately before the onset of screen 2, the triangle reached its position within screen 2, and the grid and circle were also presented. There was no updating between screens 2 and 3 as this would have required further changes between experiments, which we wanted to avoid in order to single out the updating of the layout itself. Between screens 2 and 3, the normal white screen was presented as in the other experiments. Screens 1 and 2 were presented for 2000 ms each, so participants did not self-determine presentation times. Except for updating, all methods were the same as in Experiment 1. As updating was only possible until screen 2, we expected participants to integrate in the later reference frame of screen 2. With negligible transformation costs from screen 1 to screen 2 due to updating this yields the following pattern: all same, 2 & 3 < 1 & 2, 1 & 3, all different.

Nine women and twelve men participated in Experiment 3. On average, they were 21.8 years old (*SD* = 2.6 years). 14 out of the 21 participants participated also in Experiment 1 seven only participated in Experiment 3. As results were basically identical, only the pooled data are reported.

### Results

#### Early, late, and acting reference frames

As shown in Fig 4 (third row, right side), the mean latencies differed as a function of which screen orientations matched, *F*(4, 1110) = 4.03, *p* = .003, η_p_^2^ = .13. As predicted by integration in the later reference frames, planned pairwise comparisons showed that participants acted quicker in the “all same” and “2 & 3 same” conditions compared to in other conditions, *F*s > 3.94, *p*s < .049, η_p_^2^ > .09.

For latency, there was an interaction between orientation match and rotation center, *F*(4, 1110) = 6.49, *p* < .001, η_p_^2^ = .21, which did not change the main effect orientation match. In conditions with rotation between screens, participants reacted quicker when the rotation was around the layout rather than around the screen, *F*(1, 648) = 9.53, *p* = .002, η_p_^2^ = .33. However, without rotation (i.e., screens 1 and 2 had the same orientation), participants reacted quicker when the layout moved between screens compared to when it was presented at the same location, just as in Experiment 2, *F*(1, 442) = 8.7, *p* = .003, η_p_^2^ = .35.

#### Layout intrinsic reference frame

As in prior experiments, there was a strong effect of layout orientation during testing on latency, *F*(3, 1116) = 72.1, *p* < .001, η_p_^2^ = .66. When the rectangle was presented upright, participants reacted quicker compared to when the rectangle pointed to the left or right, *F*s > 5.86, *p*s < .017, η_p_^2^ > .12, which was quicker than when the rectangle pointed downwards, *F*s > 83.7, *p*s < .001, η_p_^2^ > .67. Participants again strongly relied on layout-intrinsic reference frames.

### Discussion

With the visual updating between presentations, the participants of Experiment 3 integrated in the reference frame of the later presentation. When screen 3 matched this orientation, participants acted quicker compared to when orientations differed and participants had to mentally rotate the integrated layout into the test orientation. The visual updating largely eliminated transformation costs from screens 1 to 2, as there was no advantage if both screens had the same orientation. Integration in earlier or acting reference frames would have predicted different results.

In prior integration studies, participants updated spatial information into the reference frame from which they acted [3]. This was not possible in the present experiment. However, consistent with these results, participants updated as far as possible.

The present results differ from an experiment conducted by Avraamides and colleagues [11]. In their experiment, participants learned two layouts consisting of four objects surrounding the participant. Between presentations, participants rotated on the spot and could thus update spatial locations. Afterwards, they conducted judgments of relative direction. Results suggest that participants did not integrate the layouts during presentation, but kept separate memories and always integrated within the reference frame of the layout mentioned first during the testing (i.e., its learning orientation). Different experimental circumstances were likely responsible for the different results. In the present experiment and prior experiments that showed integration in the updated reference frame [3], the participants knew they were required to integrate spatial information, but maybe this was not the case in the experiment of Avraamides and colleagues [11]. Additionally, other studies showed that participants do not necessarily integrate spatial information spontaneously during learning, but only when given an incentive to do so [12,14]. Further differences include reliance on long-term versus short-term memory, a layout surrounding a participant versus one being presented on a 2D screen, as well as using judgments of relative direction versus having visible reference object(s). Future experiments must clarify the exact reasons.

Contrary to Experiment 2, indications for late reference frames were found in latency and not error rate. Error rates were very low; about half as high compared to the self-paced learning in Experiment 2, and even smaller compared to the baseline of Experiment 1. A floor effect may have stopped the differences in errors.

The indications for using earlier reference frames for integration in Experiment 2 were only obtained with the participants trained in the task. In Experiment 3, we used trained and naïve participants and observed identical patterns. Therefore, we cannot make inferences about task familiarity as a precondition for the influence of updating.

When the screen orientations differed between screens 1 and 2, rotations around the layout yielded quicker reactions in screen 3 compared to the rotations around the screen. However, without rotations, participants were quicker when the layout moved compared to when it stayed constant, which was a surprising pattern also found in Experiment 2. In Experiment 2, quicker reactions after the translated rather than static layouts could have been a consequence of the longer presentations times. However, this explanation cannot hold true for Experiment 3 in which presentation times were equal. Another explanation assumes memorizing not only the layout, but also the adjacent boundary, which is a highly salient cue. A boundary in a constant position relative to the layout after the screen rotations and with a static layout may be memorized more strongly alongside a layout. A layout involving a boundary located at different sides during presentation as is the case after layout rotations and after translating from one boundary to another boundary may be memorized more independently. There was no boundary around the layout during testing. The learning situations may have interfered more strongly when the boundary was memorized alongside the layout and was missing during test compared to when the layout was memorized more independently of the border when each layout side was borderless during learning at least once. The later case may have yielded quicker reactions as observed. Future experimentation must examine this speculation in more detail.

In Experiment 3 participants updated information from the first presentation to integrate in the reference frame of the later presentation. However, they did not know beforehand from which orientation they used the integrated information. Experiment 4 examined the opposite case: participants knew beforehand in which reference frame to act, but could not update the spatial information.

## Experiment 4: Acting in the Same Orientation

In prior experiments showing integration in the acting reference frame, the participants knew beforehand in which orientation they acted afterwards [3]. In Experiment 4, we examined whether such knowledge changed the reference frames used in the present setup in the same way.

### Methods

We constructed new trials in the same way as described in Experiment 1, except for the following deviations. In screen 3, the rectangle always pointed in the same direction for all 60 trials. We constructed 4 sets of 60 trials, each with the rectangle either pointing up, down, left, or right. Participants were explicitly told that the rectangle would point in that direction and also trained the experiment on these trials with a minimum of 10 trials conducted. We expected participants to integrate within the reference frame of screen 3. As described in Experiment 1 such integration yields the following pattern: all same < 1 & 3, 2 & 3 < 1 & 2, all different.

Five participants were randomly assigned to each orientation group. Nine women and eleven men who averaged 20.7 years old (*SD* = 2.0 years) participated. Two additional participants were not more accurate than the chance level and therefore were not included in the analysis.

### Results

#### Early, late, and acting reference frames

As shown in Fig 4 (bottom row), the mean error rates, *F*(4, 1171) = 6.64, *p* < .001, η_p_^2^ = .19, and latency, *F*(4, 1017) = 4.98, *p* = .001, η_p_^2^ = .15, differed as a function of which screen orientations matched. As predicted, by integration in the reference frame within which to act, planned pairwise comparisons showed that performance in the “all same,” “1 & 3 same,” and “2 & 3 same” were either more accurate or quicker compared to the other conditions, *F*s > 6.65, *p*s < .011, η_p_^2^ > .20. Better performances for “all same” compared to “1 & 3 same” and “2 & 3 same” were also predicted, but was not established, *F*s < 2.69, *p*s > .102, η_p_^2^ < .09.

#### Layout intrinsic reference frame

Contrary to the preceding experiments (Experiments 1–3), the orientation of the layout was always constant during testing in screen 3 and this orientation varied between participants. No effect of a layout intrinsic reference frame could be established. However, when looking only at between-participants effect sizes, the η_p_^2^ = .23 of Experiment 4 was comparable to the effect sizes in Experiment 1–3, which varied between η_p_^2^ = .12 and η_p_^*2*^ = .27.

#### Comparison between experiments

Thus far, strategy selection had been examined by within-experiment comparisons. In order to strengthen the argument that these patterns differ reliably between experiments, we compared the patterns between experiments by adding the factor experiment to our previous analyses resulting in a 4 (experiments) x 5 (orientation match) x 2 (rotation center) linear mixed model analysis. The patterns of the orientation match conditions were different between experiments as indicated by the interaction of the orientation match by experiment found for both error rates, *F*(12, 5883) = 2.67, *p* = .001, η_p_^2^ = .04, and latency, *F*(12, 4922) = 3.31, *p* < .001, η_p_^2^ = .07. The orientation match patterns in Experiment 1 differed from those of all other experiments in latencies, *F*s > 3.46, *p*s < .009, and by trend in errors, *F*s > 2.13, *p*s < .075. Our experimental variations reliably changed the reference frames used for integration from the baseline of Experiment 1. More importantly, orientation match patterns also differed pair-wisely between Experiment 2, 3 and 4. For Experiment 2 and 3 this was the case both for error, *F* = 3.60, *p* = .006, and latency, *F* = 3.40, *p* = .009. In Experiment 2, differences between orientation match conditions showed up in errors (Fig 4). This error pattern differed from the error pattern of Experiment 4, *F* = 4.29, *p* = .002. In Experiment 3, differences were observed in latency. There was a trend for that pattern to differ from the latency pattern of Experiment 4, *F* = 2.03, *p* = .087. When limiting the comparison to conditions in which different theoretical predictions were made between Experiments 3 and 4 (i.e., 1 & 2 same, 1 & 3 same, 2 & 3 same), the interaction was significant as well, *F* = 3.79, *p* = .023. In Experiment 2 participants profited when screen 1 and 3 were parallel, in Experiment 3 when screen 2 and 3 were parallel, and in Experiment 4 participants profited from both. The difference between these two conditions changed accordingly between experiments. The advantage of “1 & 3 same” over “2 & 3 same” in Experiment 2 reversed in Experiment 3 both for error, *F* = 6.89, *p* = .009, and latency, *F* = 9.60, *p* = .002. The Experiment 2 accuracy advantage of “1 & 3 same” leveled out in Experiment 4, *F* = 11.0, *p* < .001. Similarly, the Experiment 3 speed advantage for “2 & 3 same” also leveled out in Experiment 4, *F* = 7.98, *p* = .005. We conclude that orientation match conditions and, therefore, integration within the early, late, or acting reference frames differed reliably between experiments. The factors familiarity, updating, and knowing from where to act resulted in different integration strategies.

We also compared average performance between experiments as an indicator of the difficulty level in each experiment. We observed a difference in latency *F*(3, 95) = 15.2, *p* < .001, η_p_^2^ = .31, and a trend in errors, *F*(3, 97) = 2.33, *p* = .079, η_p_^2^ = .07. In Experiment 2 and 4 participants reacted more quickly, in Experiment 3 more accurately than participants in Experiment 1, *F*s > 5.36, *p*s < .025. We observed also lower latencies in Experiment 4 as compared to Experiment 2 and 3, *F*s > 8.86, *p*s < .006. By trend participants reacted more quickly in Experiment 2 than in Experiment 3, *F*(1, 41) = 3.65, *p* = .063.

We also observed a three-way interaction between the orientation match condition, experiment, and rotation center in latency, *F*(12, 4919) = 1.78, *p* = .045, η_p_^2^ = .04. This interaction did not change the pattern described before. It suggests that the interaction of orientation match and rotation center described in Experiments 2 and 3 was strongest in these two experiments, but less so in Experiment 1 and was not present in Experiment 4.

### Discussion

A pattern was observed in which integrating within the acting reference frame predicted better performance when presentations coincided with the acting reference frame compared to when they did not. However, performance should be even better when both, rather than only one, of the presentations coincided. This difference was predicted, but not observed. This may have been due to a floor effect. Error rates and latencies were very low in these conditions compared to the other experiments. At this level, the task might have been so easy that it did not differentiate reliably. More importantly, none of the other predictions were for the observed pattern of the results (i.e., the significant differences observed); the results are inconsistent with integration in earlier or later reference frames. Therefore, we conclude that the way that participants integrated in the reference frame of acting was similar how they integrated in the prior experiments [3]. As this average pattern was only observed when the participants knew beforehand in which orientation they would act later, although not in Experiments 1–3, we also conclude that this knowledge was highly crucial for integration in the acting reference frame.

The results might have been even stronger when participants not only knew the acting reference frame, but were also able to update into this reference frame. However, as noted in the discussion for Experiment 3, this was difficult to realize while still trying to keep the experiments as similar as possible. In addition, the point made was that both updating and knowing within which reference frame to integrate individually influenced the reference frame used for integration.

The predicted differences were partly found in latency. This suggests that not all transformations into the acting reference frame were conducted before the onset of screen 3. Some of these transformations might happen within screen 3; otherwise, no latency difference would have been observed.

The pattern of results was distributed across latency and errors. Nevertheless, the numerical pattern was highly similar. Therefore, we do not think that there was a specific tradeoff between the two measures.

In Experiment 4, the pattern of results was spread across latency and errors; in Experiment 2, it was found in errors only; in Experiment 3, in latency only. While the patterns differed significantly between experiments, it is unknown why effects were observed sometimes in errors, latencies, or both. It may be that participants changed their response behavior. In Experiment 2, participants self-paced their presentation time. They may have adopted a constant individual rhythm of looking then responding. When keeping the response time largely constant, differences might have mainly shown up in error. However, in Experiment 3, participants could have focused on accuracy. Participants were at least numerically the most accurate in Experiment 3. Such a focus on accuracy could have yielded a floor effect, with differences showing up only in latency. However, we do not know what would have caused a switch in response strategy, as all participants in all experiments were instructed to react as accurately and quickly as possible.

Could the different patterns of results have originated from general difficulty levels between experiments? Compared to the baseline of Experiment 1 all variations (familiarity, updating, knowing from where to act) made the task easier for the participants. Also participants’ reaction times differed between Experiment 2, 3 and 4. When knowing from where to act (Experiment 4) participants reacted more quickly than in Experiment 2 and 3. Knowing in which orientation to act participants could prepare their reaction already within screen 2. This might have boosted their reaction times. However, differences in integration strategy might also originate from differences in task difficulty. Integration within the reference frame to act might be a strategy applied for very easy integration tasks in general, not only when knowing beforehand from where to react. Future experimentation has to clarify this possibility.

Contrary to Experiments 1–3, we did not observe evidence supporting layout-intrinsic reference frames. In Experiments 1–3, the effect was within-participants (i.e., each participant was tested in all layout orientations); however, in Experiment 4, it was between participants (each participant only experienced one layout orientation during the test) and it was numerically present in latency, but was not significant. However, the between-participants effect sizes in Experiment 4 were comparable in size to the between participant effect sizes in Experiment 1–3. Consequently, we think that the lack of effect for intrinsic reference frames was primarily due to the lower power when testing between, rather than within, participants.

## General Discussion

Despite its relevance for everyday navigation, surprisingly little is known about spatial integration across separate experiences, such as a document presented piecewise on a screen, opposing views of a room, or multiple rooms or streets. Our results show that participants can flexibly adjust their integration strategy to the properties of the task and their experience; the reference frames used for integration rely on intrinsic layout organization (Experiment 1–3) and differ as a function of task familiarity and available learning time (Experiment 2), the possibility of updating the orientation of a layout between experiences (Experiment 3), and the knowledge of the retrieving situation (Experiment 4). As a consequence, participants respectively prioritize the reference frame when earlier information was presented, the later reference toward their updated orientations, and the reference frame from which the task was conducted. Results replicate earlier results from 3D environments on a 2D screen. Furthermore, they show that contextual influences indeed can change integration strategy, thus providing an explanation for seemingly contradictory results in prior experimentations.

Participants used earlier reference frames for integration as well as salient reference frames intrinsic to the layout itself. This is in line with prior experimentation [7–10], but also generalizes it to different circumstances. First, it generalizes from integrating successively added information (i.e., learning layout 1, then layouts 1 and 2 together) to the case of integrating separate experiences (learning layout 1 separately from layout 2). Second, it generalizes prior results from long-term memory coding to working memory processing. In prior experiments, participants learned layouts in the order of minutes and then accessed information from a remote place, which suggests long-term storage. In the present experiments, participants learned in the order of seconds and retrieved the information immediately afterwards, which can be solved based on working memory. Finally, it also generalizes the effect from learning 3D objects in a room to the 2D case of learning objects on a screen. Effects of early and salient reference frames generalize across a wide variety of situations.

Participants in Experiment 2 preferentially used reference frames of earlier presentations to integrate with information presented later. These results were only found when participants trained in the task were granted as much time for integration during presentation as they wanted. Using earlier reference frames for integration seems appropriate when time is no problem. In the aforementioned studies, earlier reference frames were not used when a salient reference frame, such as the main orientation of a room or the to-be-learned object layout, was present [1,7,8]. The results of the present experiments also showed a strong influence of layout intrinsic reference frames. However, here, intrinsic layout orientations were decoupled from earlier or later reference frames. Both effects were observed in parallel.

Participants’ integration strategies were not limited to intrinsic or earlier reference frames. As shown in Experiment 4, knowing from where to act afterwards was sufficient for integrating in this reference frame, thus specifying prior results on spatial integration where updating was also possible [3]. Without knowing from where to act, the results of Experiment 3 showed that visual updating to the second presentation yielded integration in this later reference frame. The present results extend prior results in showing that both factors—updating and knowing from where to act—can trigger a certain integration strategy. Furthermore, updating and knowing from where to act do not only influence spatial integration within a 3D environment, but also work with 2D screen presentation. Both the earlier work [3] and present experimentation rely on working memory. It is an open question as to whether knowing from where to act as well as updating will influence long-term memory content in the same way or whether initial or salient orientations will dominate in these cases.

In the experiments, the intrinsic orientations of the rectangle and triangle were always parallel and participants in most of the cases heavily relied on this layout-intrinsic reference frame. Layouts with less prominent intrinsic reference frames may guide participants to rely less strongly on layout intrinsic reference frames. This might be attained by changing the relative position of the triangle relative to the rectangle in each trial. For example, the rectangle points upwards, but the triangle to the right; then, participants must additionally learn this relative orientation between rectangle and triangle in order to solve the task. Such a task might be too demanding for many participants; therefore, we did not use it. Further investigations are needed to examine this issue.

In the present experiments, all information was presented on a screen providing a constant frame within each experienced view similar to a car windshield. This situation was identical in all conditions and cannot account for the differences found. Our results might be limited to situations providing such a constant environmental reference frame; however, we do not think this is very likely as experiments of learning locations within a room without such a frame show comparable effects as noted before [3,7–10].

Spatial integration does not only happen at the level of views. Spatial information is also integrated across gazes to accumulate information within a view. Is such behavior flexible? While whole visual fields of gazes are surely not integrated automatically [16], adaptations to line orientation, form, or faces persist across gazes [17], thus suggesting automatic updating of certain attended features across gazes. Integration across gazes might be more automatic and less flexible than integration across views as in the present experiments.

Will strategic flexibility occur at all time scales? In the integration of overlay stimuli, the grid cell not occupied in two successive presentations must be identified. Very short presentations times result in one percept making the task trivial [20]. Separately perceived stimuli will be integrated better the more presentation time suffices for encoding into visual short-term memory [22]. Here, strategic variations become possible [25] as in the present study. The underlying working memory processes enabling such flexibility—for example, reference frame transfers—are not yet understood.

Our results showed how certain circumstances influence the reference frame of spatial integration, thus resolving inconsistencies between prior studies. They generalize findings obtained with different methods from the 3D case of learning locations within a room to the 2D case of learning layout parts on a screen, thus showing the general nature of spatial integration processes. Furthermore, our results disentangle previously intermingled issues, namely salient and established reference frames, as well as updating and knowing from where to act. Most importantly, by integrating different approaches into one common methodology we could show that there are unique circumstances capable of changing the reference frames used for integration.

## Conclusions

Spatial integration of separately experienced information is an everyday task that has only just started to be examined. Our results suggest that participants can flexibly adjust the reference frame used for spatial integration to the way they perceived, acted, and the actual structure of the integrated layout.

## Supporting Information

S1 Dataset Data from all experiments. Lines correspond to trials. Time is measured in ms. In the eye-tracking data “1” means fixating the rectangle (“trigger”), “2” fixating the triangle (“context”), “3” fixating the circle (“target”), and “9” fixating somewhere else.
(CSV)
